# Supplementary figures and images for: Egg excretion indicators for the measurement of soil-transmitted helminth response to treatment
Source: PLoS Negl Trop Dis. 2022 Aug 2;16(8):e0010593. doi: 10.1371/journal.pntd.0010593 (PMC9374261; doi:10.1371/journal.pntd.0010593)

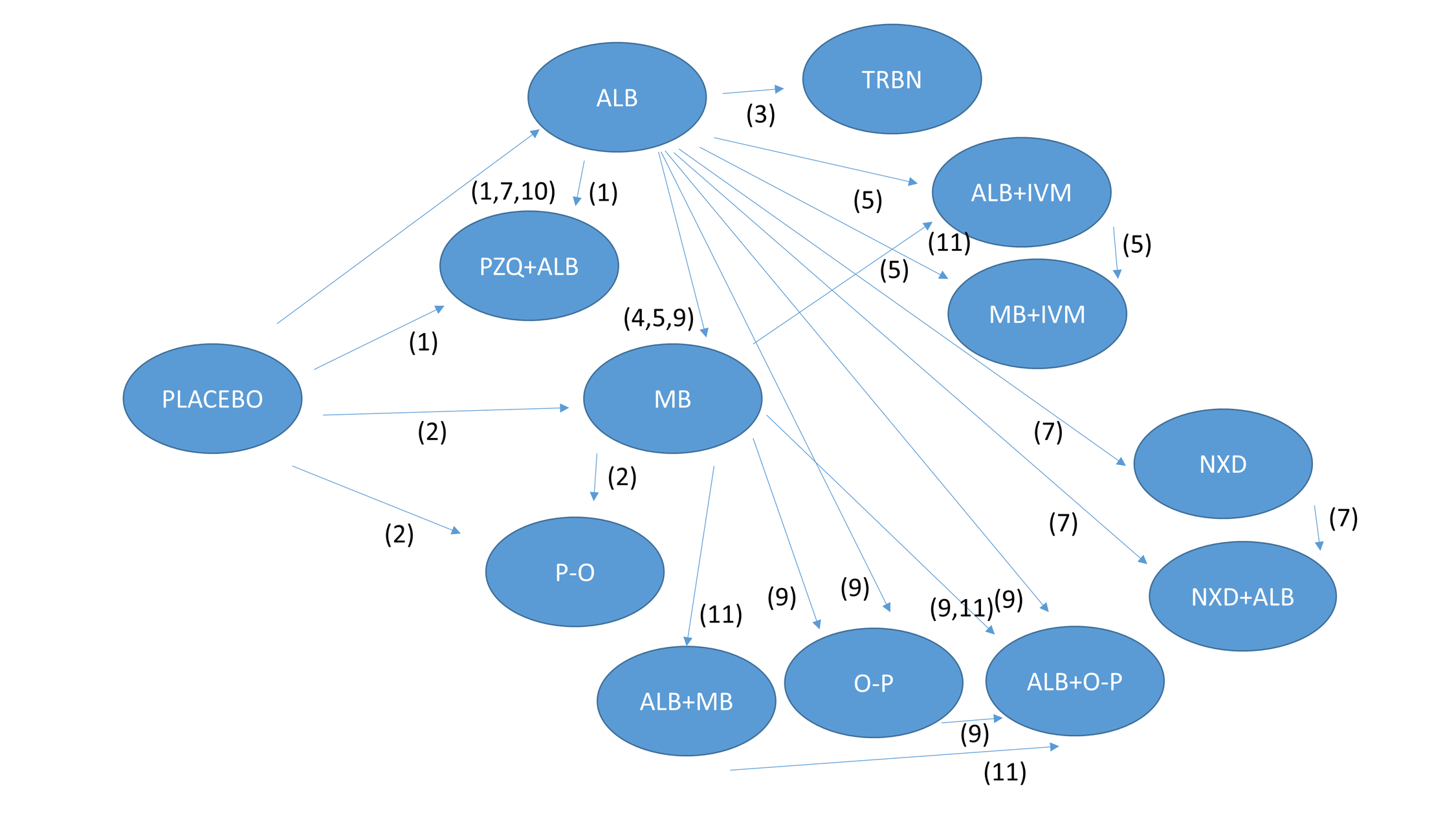

Supplement: S1 Fig — (TIF) [file pntd.0010593.s008.tif]
