# Supplementary material for: Egg excretion indicators for the measurement of soil-transmitted helminth response to treatment
Source: PLoS Negl Trop Dis. 2022 Aug 2;16(8):e0010593. doi: 10.1371/journal.pntd.0010593 (PMC9374261; doi:10.1371/journal.pntd.0010593)
Supplement: Attachment — Submitted filename: Olliaro-Vaillant et al_STH-drug response_ResponseToReviewer.docx [file pntd.0010593.s009.docx]

PNTD-D-22-00001 "**Egg excretion indicators for the measurement of soil-transmitted helminth response to treatment**" – Point-by-point response

Oxford, Strassen and Basel, 3 June 2022

Dear Drs De'Broski and Aysegul Taylan,

Thank you very much for your e-mail dated 22 April 2022 in connection to the aforementioned manuscript. We have now revised our piece in the light of the reviewers’ suggestions.

Below, please find our point-by-point response, indicating how and where in the manuscript changes have been made. As requested, we enclose two versions of our revised manuscript: (i) a track-changed version and (ii) a final clean version. Please note that the line numbers indicated below refer to the final clean version.

We hope that we have satisfactorily addressed the points raised by the reviewers, and look forward to your decision.

Yours sincerely,

Piero L. Olliaro, Michel Vaillant and Jennifer Keiser

(on behalf of all co-authors)

*********************************************************************************

**Reviewer #1**

The paper analyzes a large dataset of data coming from 13 studies; the data analysis is very detailed however it seems to me that the study has not been conducted with any hypothesis or research question but rather conducted because of the availability of the dataset (!).

**Answer**: The aim of the present study was to identify suitable approaches to quantitate the effects and compare the efficacy of different anthelmintic treatments when administered to individuals with single or multiple species soil-transmitted helminth infections from different trials. This is clearly stated in the paper, including the Abstract. We accept that we had to make with the primary data that were available to us for this line of scientific inquiry.

The stated objective of the study (line 103) “identify suitable approaches to quantitate the effect and compare the efficacy of different anthelminthic treatments…” is rather vague and not necessarily relevant since a WHO manual is available for this purpose (cited by the authors in reference #8) and the authors out of their analysis seems not to propose any innovation to the existing methodology.

**Answer**: With due respect, we disagree with Reviewer #1. We provide in the discussion an explanation on why the WHO guidelines have limitations when evaluating drug efficacy in trials, as opposed to general surveillance. As also found in other helminthic infections^[1]^, group means quantitate the overall response of a population, and do not detect the distribution of responses and the proportion of individuals with sub-optimal responses. Therefore, we compared individual egg reductions in soil-transmitted helminth infections. Furthermore, we also explored statistical approaches to help explain variations in individual responses. These approaches are all described in the paper.

All the data reported by the authors are already well known (e.g. the different efficacy of the drug on the different STH species, the age distribution of STH infections, the importance to standardize study if we want analyze the data in combined manner, the overall shortage of viable option to treat STH….) and it seems to me that this detailed analysis to be a sterile academic exercise.

**Answer**: We agree with the Reviewer that the individual study data are known, and these questions have been raised before. However, here, we present a rigorous individual participant data meta-analysis, adding the power of numbers of individual studies. We present new evidence of heterogeneity in drug efficacy and its evaluation, age distribution, across studies, and looked into possible explanations. This is all except sterile academic exercise, as it has direct implications for guidelines and policies.

I suggesting here few research questions that could have been addressed by the authors analyzing so large and diverse set of data:

**Answer**: We thank the Reviewer for this set of ideas. We carefully considered the points offered and provide specific responses why we feel that addressing these points would amount to writing up another paper.

1- evaluating the drug efficacy (for example of albendazole against T. trichiura) in the context of the time during which the children have been previously exposed to PC (i.e. if the drug efficacy progressively decrease with the increasing number of PC round administered of it is substantially stable).

**Answer**: Unfortunately, the information was not collected in the studies used, hence, this kind of analysis is not possible with the data at hand.

2- evaluating the existence of geographical difference in term of drug efficacy (i.e. if albendazole efficacy for hookworm in Asia is constantly different from the dug efficacy in other regions) this result could support the hypothesis of differences in STH species in the different regions

**Answer**: We believe this point is taken into account in our analyses which allow for the site of the individual studies as a hierarchical factor in multivariate analyses and included country as fixed effect. No country difference was shown, even when retested in the models after being discarded by the elasticnet procedure of variable selection.

3- since some study has used 2, 3 or even 4 specimen to define prevalence and intensity of infection, it would have been very interesting to understand the contribution of the 2nd, 3rd and 4th reading on the evaluation of the drug efficacy (or prevalence and intensity of infection) and consider the advantages provided by these multiple reading in view of the additional cost needed to collect and analyze multiple specimen.

**Answer**: We agree with the Reviewer that it would be interesting to determine the effect of sampling effort on estimating drug efficacy. Yet, this is not really novel. See, for example, a study by Levecke and colleagues published in 2014 (cited by us, see reference # 15) who evaluated the prevalence, fecal egg count (FEC), cure rate (CR) and egg reduction rate (ERR) obtained from duplicate Kato-Katz thick smears on two consecutive stool samples (2×2 KK) with data from a single Kato-Katz thick smear on a single stool sample (1×1 KK), a duplicate Kato-Katz thick smear on one stool sample (1×2 KK) and a single Kato-Katz thick smear on two consecutive stool samples (2×1 KK)^[2]^. Alternatively Nikolay et al. (2014) evaluated the sensitivity and Bayesian credible intervals of 1, 2 or 3 KK and compared mean egg count with other diagnostic methods^[3]^. In view of prior existing studies, we decided not to include such an analysis.

In conclusion, with the possibility to analyze so large and diverse set of data and with the proved analytical capacities of the authors I think the paper is a missed opportunity to contribute even marginally to the knowledge on STH.

**Answer**: We agree with the Reviewer that the collection and harmonization of a large ensemble of individual participant-based data is an opportunity for further analyses. This dataset is now fully curated and indexed within the IDDO repository (see: https://www.iddo.org/) and will be available for other researchers to answer some of the proposed research questions.

*********************************************************************************

**Reviewer #2**

Preventive chemotherapy, based on the periodic use of anthelminthic drugs, either alone or in combination, is considered by WHO a public health tool against soil-transmitted helminth infections (STH). Regular deworming reduces both the morbidity caused by these infections and the occurrence of severe complications.

In the context of large scale STH control programmes, monitoring of drug efficacy and anthelmintic resistance is needed.

In this perspective, the authors, considering that different factors may influence drug efficacy making difficult to standardize treatment outcome measures, tried to identify suitable approaches to assess and compare the efficacy of different anthelmintic treatments.

They worked on a database including the results from 13 studies (11 randomized controlled trials and 2 observational studies) in which infected subjects (n=5688; 10220 infections) received single-agent or combination therapy, or placebo. The selected studies reported reduction in worm egg counts in stools calculated from before to 14-21 and 22-45 days after treatment using different methods.

Results are well presented and the large amounts of information collected in tables and figures that may require some time to interpret if not familiar with the topic. However, readers will take advantage from the effort to present the details of the great mass of information obtained from the 13 studies. The studies included subjects with single or multiple species STH infections treated with benzimidazoles (albendazole, mebendazole, alone and in combination with other drugs, in particular ivermectin and oxantel pamoate) as well as other treatments.

The individual subject and group mean response to treatment were analysed by using egg reduction rate.

The results allowed to demonstrate that a) combining the traditional efficacy assessment using group averages with individual responses provides a more complete picture of how anthelmintic treatments perform, b) most treatments analyzed fail to meet the WHO minimal criteria for efficacy based on group means, c) drug combinations (i.e., albendazole-ivermectin and albendazole-oxantel pamoate) are promising treatments for STH infections. The latter point is of particular interest as drug combinations, in addition to being more effective, can help mitigate the potential emergence of drug resistance.

In conclusion, the article is very interested and the important findings will help to optimize preventive chemotherapy programmes for STH infections and their monitoring.

**Answer**: We thank the Reviewer for this excellent summary of our work.

Editing

Line 217

The comma should be deleted

**Answer**: Done

Line 314

loer should be corrected to lower

after AL: a space is required

after A.lumbricoides the full stop should be deleted

**Answer**: We addressed these typing errors and are grateful to the Reviewer for reading our piece so carefully.

Line 504

Tropicale should be Tropical

**Answer**: Indeed!

Line 513

Organization WH is probably World Health Organization

**Answer**: Reviewer #2 is correct and we have revised accordingly.

516

ameroon should be Cameroon

**Answer**: Sorry for this typing mistake; has been addressed.

Table 1

Panama is reported first as Panamá, then Panamá. Please check

**Answer**: We carefully checked country names in Table 1 and corrected accordingly.

1. Olliaro PL, Vaillant M, Diawara A, Coulibaly JT, Garba A, Keiser J, King CH, Knopp S, Landouré A, N'Goran EK, Raso G, Scherrer AU, Sousa-Figueiredo JC, Stete K, Zhou XN, Utzinger J (2015). Toward measuring *Schistosoma* response to praziquantel treatment with appropriate descriptors of egg excretion. PLoS Negl Trop Dis. **9**(6): e0003821.
2. Levecke B, Montresor A, Albonico M, Ame SM, Behnke JM, Bethony JM, Noumedem CD, Engels D, Guillard B, Kotze AC, Krolewiecki AJ, McCarthy JS, Mekonnen Z, Periago MV, Sopheak H, Tchuem-Tchuenté LA, Duong TT, Huong NT, Zeynudin A, Vercruysse J (2014). Assessment of anthelmintic efficacy of mebendazole in school children in six countries where soil-transmitted helminths are endemic. PLoS Negl Trop Dis. **8**(10):e3204.
3. Nikolay B, Brooker SJ, Pullan RL. 2014. Sensitivity of diagnostic tests for human soil-transmitted helminth infections: a meta-analysis in the absence of a true gold standard. Int J Parasitol. **44**(11): 765-774.
